# Intra‐session repeatability of quantitative metrics using widefield optical coherence tomography angiography (OCTA) in elderly subjects

**DOI:** 10.1111/aos.14327

**Published:** 2019-12-12

**Authors:** Jimmy Hong, Bingyao Tan, Nguyen Duc Quang, Preeti Gupta, Emily Lin, Damon Wong, Marcus Ang, Ecosse Lamoureux, Leopold Schmetterer, Jacqueline Chua

**Affiliations:** ^1^ Singapore Eye Research Institute Singapore National Eye Centre Singapore City Singapore; ^2^ Department of Ophthalmology Lee Kong Chian School of Medicine Nanyang Technological University Singapore City Singapore; ^3^ School of Chemical and Biomedical Engineering Nanyang Technological University Singapore City Singapore; ^4^ Academic Clinical Program Duke‐NUS Medical School Singapore City Singapore; ^5^ Department of Clinical Pharmacology Medical University of Vienna Vienna Austria; ^6^ Center for Medical Physics and Biomedical Engineering Medical University of Vienna Vienna Austria

**Keywords:** filters, macula, optical coherence tomography angiography, perfusion density, repeatability, vessel density

## Abstract

**Purpose:**

To assess the repeatability of retinal vascular metrics using different postprocessing methods as obtained from the swept‐source optical coherence tomography angiography (SS‐OCTA).

**Methods:**

Thirty‐two participants (63% males; mean [SD] age, 70 [7] years) underwent SS‐OCTA imaging (PLEX ^®^ Elite 9000, Carl Zeiss Meditec, Inc., Dublin, USA). Each participant underwent 2 repeated scans of 2 scan protocols: a macular‐centred 3 × 3‐mm^2^ and a widefield 12 × 12‐mm^2^ for a total of 4 acquisitions. Images of superficial vascular plexuses (SVP) and deep vascular plexuses (DVP) were processed using different filters to generate the perfusion density (PD) and vessel density (VD). Vessel enhancement filters ranged from vessel targeted (Hessian and Gabor filters), classical denoising (Gaussian filter), to a scale‐selective adaption (modified Bayesian residual transform [MBRT]). Intra‐session repeatability of the different filters and their correlation with the original data set were calculated with the intraclass correlation coefficient (ICC) and Pearson's *r*.

**Results:**

Of the 32 eyes, 17 and 15 were right and left eyes, respectively. For 3 × 3‐mm^2^ scans, both MBRT and Gabor filters yielded very good repeatable PD and VD (both ICCs > 0.87) values. Gabor filter was the most correlated with the original data set for the OCTA metrics (*r* = 0.95–0.97). For 12 × 12‐mm^2^ scans, MBRT filter produced good‐to‐moderate ICC values for SVP (ICC>0.89) and DVP (ICC>0.73) metrics. Both the MBRT and Gabor filters were highly correlated with the original 12 × 12‐mm^2^ scan data set (*r* = 0.96–0.98). The ICCs for the agreement between 3 × 3‐mm^2^ and cropped 12 × 12‐mm^2^ were high only for the PD values at the SVP layer and were poor for the VD at SVP and DVP measurements (ICC < 0.50).

**Conclusion:**

Our findings show that with the proper choice of postimaging processing methods, SS‐OCTA metrics can be obtained with high repeatability, which supports its use in various clinical settings.

## Introduction

Imaging the retinal vasculature plays a crucial role in the management of a variety of retinal diseases (Chung et al. 2018; Johannesen et al. 2019) such as diabetic retinopathy (Ting et al. 2017) and age‐related macular degeneration (Cicinelli et al. 2017). Optical coherence tomography angiography (OCTA) can non‐invasively provide vasculature information about the retina using the decorrelation signal between repeated scans, allowing it to be used in a larger group of patients (Kashani et al. 2017; Ang et al. 2018; Chua et al. 2018a,b,^2019^). Furthermore, OCTA provides depth‐resolved imaging and thus allows for distinction of the superficial and deep retinal vasculature (Spaide et al. 2018). Before the OCTA can be established as a cornerstone of retinal vascular imaging, it is crucial to understand the factors that can affect its measurement variability.

Precision of vessel density measurements from OCTA data can vary by sessions, technicians (alignment, stability, focus), subjects (motion, optical clarity), systems (e.g. acquisition speed, resolution) and image processing routines (e.g. registration, segmentation) (Lozano & Twa 2012). Despite these potential hurdles, excellent repeatability and reproducibility of OCTA vessel density measurements have been reported in normal (Lei et al. 2017) and patients with retinal diseases (Czako et al. 2018; Lee et al. 2019). Others have also evaluated the reproducibility of retinal vascular metrics using different OCTA instruments (Corvi et al. 2018) and binarization methods (Shoji et al. 2018). However, these studies have mostly analysed smaller scan dimensions, ranging from 3 × 3‐mm^2^ (Corvi et al. 2018; Czako et al. 2018; Lee et al. 2019) to 6 × 6‐mm^2^ (Lei et al. 2017; Takusagawa et al. 2017; Chen et al. 2018). A relatively limited field of view makes it unsuitable to evaluate peripheral areas of capillary dropout or peripheral neovascularization in diabetic patients. This issue can be partially overcome by using widefield scans of 12 × 12‐mm^2^. Therefore, the repeatability of quantitative metrics from widefield OCTA using various postimage processing methods must be assessed before these data can be confidently interpreted in clinical research and practice.

The purpose of the study was to assess the intra‐session repeatability of quantitative retinal vascular metrics using postimage processing methods obtained using the swept‐source optical coherence tomography angiography (SS‐OCTA; PLEX Elite 9000 prototype; Carl Zeiss Meditec, Inc.; version 1.6).

## Materials and Methods

### Study population

We conducted a cross‐sectional study from January to July 2018 on 32 participants who were consecutively recruited from a population‐based study under the ‘PRevention Of and InterVentIon for eye Diseases in the Elderly’ (PROVIDE) programme. Briefly, PROVIDE is a population‐based study of 650 Singaporeans aged 60 and above with participants selected from a computer‐generated list stratified by age and ethnicity, with 50% Chinese, 25% Malays and 25% Indians. Participants were excluded from study if they were incarcerated, bedridden or uncontactable via phone calls and home visits. Informed consent was obtained from all individual participants included in the study. All procedures performed in studies involving human participants were in accordance with the ethical standards of the SingHealth Centralised Institutional Review Board (IRB) and with the 1964 Helsinki declaration and its later amendments or comparable ethical standards.

### Clinical evaluation

Each participant underwent a thorough ocular and systemic assessment and was administered questionnaires to evaluate their cognition and socio‐demographic status. Participants were assessed for their visual acuity, ocular biometry, intra‐ocular pressure (Chua et al. 2018a,b,^2019^) and ocular health status (Chua et al. 2015). Their pupils were dilated with 1% tropicamide and 2.5% phenylephrine hydrochloride before they underwent fundus photography, OCT and SS‐OCTA. We also identified eyes with the presence of age‐related macular degeneration (Kawasaki et al. 2008), glaucoma (Shen et al. 2008) and retinopathies (Wong et al. 2008).

### Swept‐source optical coherence tomography angiography imaging

One eye was randomly selected from each participant for OCTA imaging with a SS‐OCTA system. The device has a central wavelength of 1060 nm, bandwidth of 100 nm, optical axial resolution of 6.3 *μ*m in tissue and acquisition rate of 100 000 A‐scans per second. FastTrac motion correction software based on linescan ophthalmoscope was used whenever possible to minimize motion artefacts.

During a single clinic session, each patient received four acquisition scans, where two repeated scans of macula centred 3 × 3‐mm^2^ scans and widefield 12 × 12‐mm^2^ scans that visualized the optic nerve head, macula and peripheral retina were taken. Each 3 × 3‐mm^2^ scan was captured with 300 × 300 sampling (i.e. 300 A‐scans per B‐scan with 300 B‐scan positions) while each 12 × 12‐mm^2^ scan was captured with 500 × 500 sampling. A time interval of approximately 5 min was given between the two repeated scans, and if a scan was deemed to be grossly inadequate due to issues such as significant motion artefacts or misalignment by the technician, it was excluded from the analysis and a replacement scan was taken. Between scans, artificial tears were applied whenever necessary. Optical coherence tomography angiography (OCTA) images were generated by an optical microangiography protocol (Wang et al. 2010; An et al. 2011), each scan was automatically segmented into the superficial (SVP) and deep vascular plexus (DVP), and projection artefacts were automatically removed from the DVP by the PLEX Elite Review Software v1.6.0.21130. The SVP is taken to be from the inner limiting membrane (ILM) to the inner plexiform layer (IPL) while the DVP is defined to start from the IPL to the outer plexiform layer (OPL); the IPL boundary is calculated as 70% of the distance from the ILM to the OPL while the OPL boundary is defined as 110 *μ*m above the retinal pigment epithelium. If necessary, manual correction of automated segmentation outputs was carried out to ensure an accurate segmentation. All scans were performed in the same sequence by a trained ophthalmic technician.

**Figure 1:**
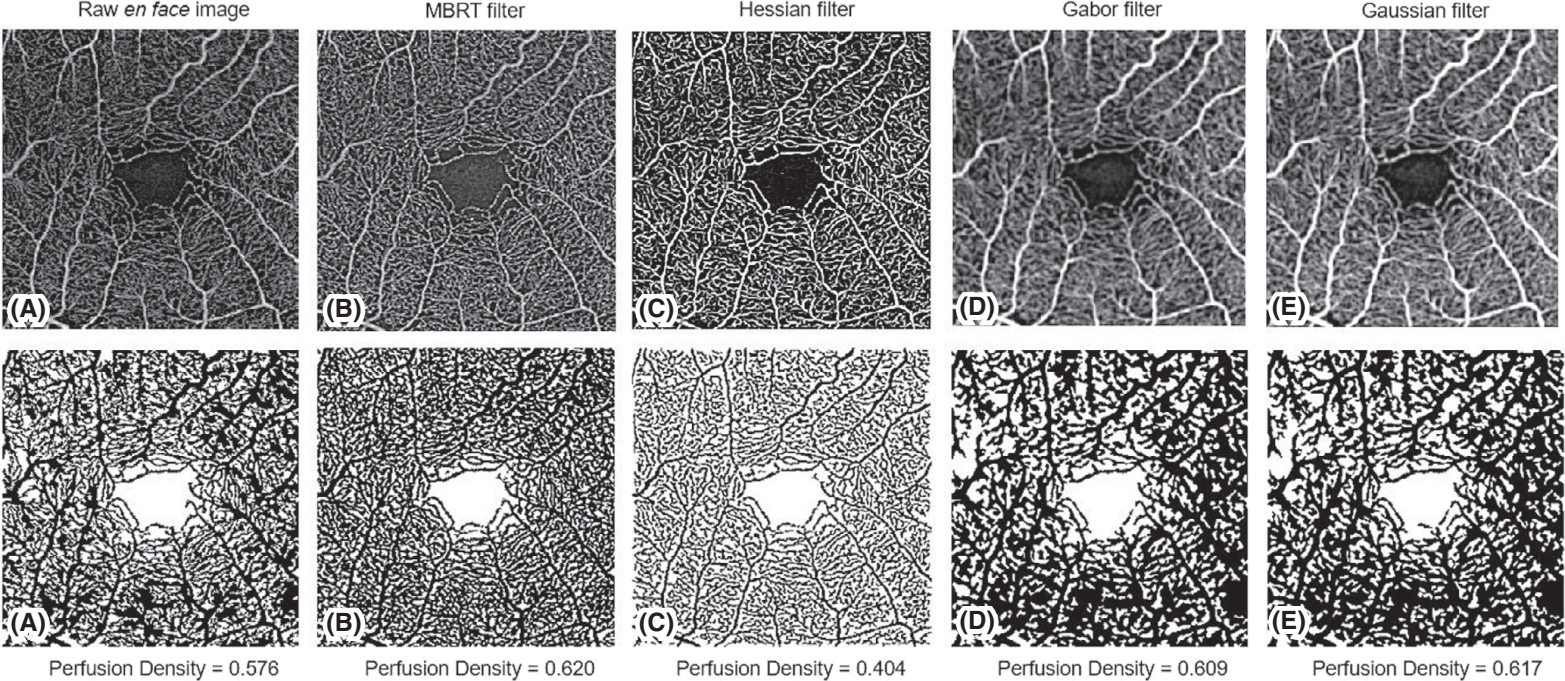
Application of various filters to a 3 × 3‐mm^2^ scan of the superficial vascular plexus and their corresponding perfusion densities.

**Figure 2:**
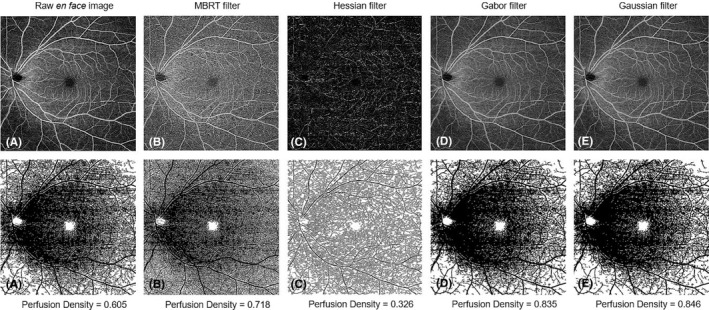
Application of various filters to a 12 × 12‐mm^2^ scan of the superficial vascular plexus and their corresponding perfusion densities.

**Figure 3:**
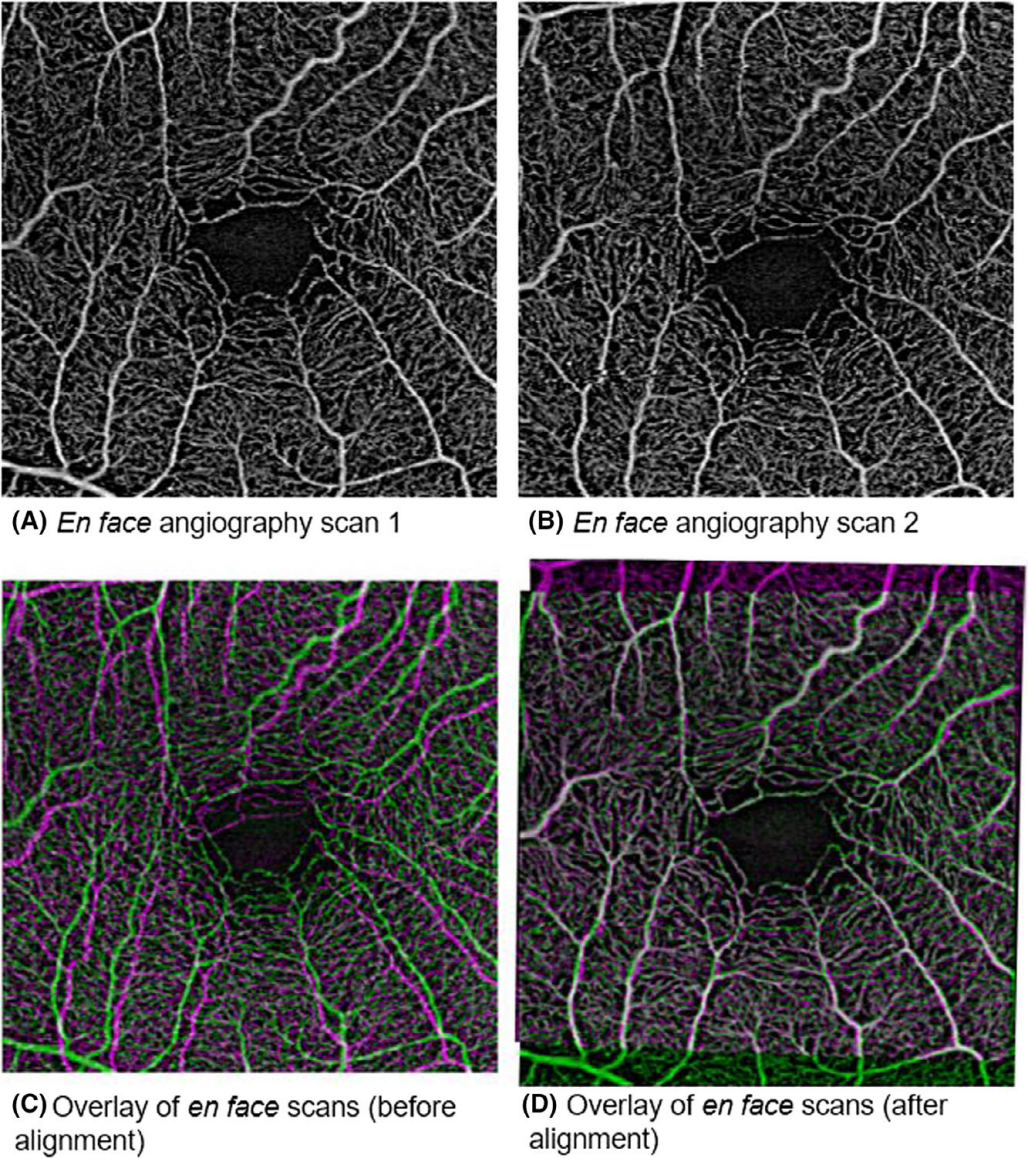
Overlay of 2 *en face* scans showing sampling of different vessels of the same eye.

The *en face* scans of the SVP and DVP of the 3 × 3‐mm^2^ and 12 × 12‐mm^2^ scans were exported from the PLEX Elite Review Software for calculation of the perfusion density (PD), defined as the total perfused area per unit area, and vessel density (VD), defined as total length of perfused vasculature per unit area, of the SVP and DVP. The main difference between PD and VD is that PD accounts for vessel calibre (Figures 1 and 2) while VD treats all vessels equally. To account for varying scan centration, each pair of *en face* angiography scans was aligned in with MATLAB (MathWorks Inc., Natick, MA) using the ‘imregtform’ function that estimates a geometric transformation to optimally align two images and the overlapping regions of each scan were cropped for further analysis (Figure 3). Next, a filter was applied to enhance the vascular structures. A total of 4 filters were tested: (1) a modified Bayesian residual transform (MBRT)‐based filter (Tan et al. 2018); (2) a Hessian filter (Frangi et al. 1998); (3) a Gabor filter; and (4) a Gaussian filter with a standard deviation of 3 pixels. Lastly, each enhanced image was binarized by mean values to obtain its corresponding vascular density values. The processing time of the filtering choices per scan varied, where it was the quickest without any filter (<0.01 seconds), Gaussian filter: 0.02 seconds, Hessian filter: 0.67 seconds, Gabor filter: 1.23 seconds and longest with the MBRT filter: 2.27 seconds on a desktop with i9‐9700 × CPU and 64GB RAM.

### Statistical analyses

The repeatability of the vascular metrics was assessed using intraclass correlation coefficients (ICC). Intraclass correlation coefficient (ICC) values less than 0.5, between 0.5 and 0.75, between 0.75 and 0.90 and greater than 0.90 indicate poor, moderate, good and excellent repeatability, respectively (Koo & Li 2016). Additionally, the data sets obtained using various postimage processing algorithms were correlated with the corresponding *en face* image (without any filter) by calculating the different values of Pearson's *r*. All statistical analyses were performed using R version 3.3.1.

## Results

A total of 32 randomly selected eyes from 32 patients were included in the study of repeatability of 3 × 3‐mm^2^ and 12 × 12‐mm^2^ OCTA vascular metrics. Table 1 reports the demographics and clinical characteristics of these participants where the mean (SD) age was 70 (7) years, 63% males and 69% Chinese. Their LogMAR visual acuity was 0.12 (0.15) (Snellen acuity = 6/7.5^−2^) and spherical equivalent was + 0.04 (1.99). Two thirds of the participants were phakic. A minority of the eyes were diagnosed with ocular diseases: age‐related macular degeneration (*n* = 1), glaucoma (*n* = 1) or diabetic retinopathy (*n* = 1). The OCTA's signal strength index (out of 10) of 3 × 3‐mm^2^ scans was 7.9 (0.6) while that of the 12 × 12‐mm^2^ scans was 7.6 (0.6).

**Table 1:** Clinical and Ocular characteristics of Participants (*n* = 32 Participants).

Characteristics	Mean (SD), *n* (%)
Clinical characteristics	
Age	70 (7)
Sex, male	20 (63%)
Ethnicity, Chinese	18 (69%)
History of systemic diseases	
Diabetes mellitus	12 (38%)
Hypertension	19 (59%)
Dyslipidaemia	18 (56%)
Ocular characteristics	
Imaged eye	
Right	17 (53%)
Left	15 (47%)
Lens status	
Phakic	20 (63%)
Pseudophakic	12 (37%)
Aphakic	0
Distance Visual Acuity LogMAR	0.12 (0.15)
Spherical equivalent	+0.04 (1.99)
History of significant ocular disease	
Age‐related macular degeneration	1 (3%)
Glaucoma	1 (3%)
Diabetic retinopathy	1 (3%)

CI = confidence interval, LogMAR = logarithm of the minimum angle of resolution, SD = standard deviation.

### Mean, repeatability and correlation analysis of 3 × 3‐mm^2^ vascular parameters

The mean PD and VD values of the SVP and DVP of the analysed 3 × 3‐mm^2^ scans, their corresponding ICC values and their correlation with the raw *en face* scan are shown in Table 2.

**Table 2:** Mean, intraclass correlation coefficient values and correlation analysis of 3 × 3‐mm^2^ vascular parameters.

Characteristics	A) Mean (SD)	B) ICC (95% CI)	*P* value	C) Pearson's *r* (95% CI)	*P* value
Superficial vascular plexus					
Perfusion density					
1. Raw *en face* scan	0.449 (0.018)	**0.88 (0.75, 0.94)**	<0.001	Reference	
2. MBRT filter	0.452 (0.015)	**0.90 (0.79, 0.95)**	<0.001	**0.95 (0.90, 0.98)**	<0.001
3. Hessian filter	0.298 (0.037)	**0.80 (0.59, 0.90)**	<0.001	0.60 (0.31, 0.78)	<0.001
4. Gabor filter	0.451 (0.021)	**0.88 (0.75, 0.94)**	<0.001	**0.97 (0.94, 0.99)**	<0.001
5. Gaussian filter	0.444 (0.024)	**0.88 (0.76, 0.94)**	<0.001	**0.91 (0.82, 0.96)**	<0.001
Vessel density (mm^−1^)					
1. Raw *en face* scan	0.066 (0.004)	**0.85 (0.70, 0.93)**	<0.001	Reference	
2. MBRT filter	0.038 (0.001)	**0.89 (0.76, 0.94)**	<0.001	**0.79 (0.62, 0.89)**	<0.001
3. Hessian filter	0.060 (0.006)	**0.82 (0.63, 0.91)**	<0.001	**0.83 (0.68, 0.91)**	<0.001
4. Gabor filter	0.059 (0.004)	**0.83 (0.65, 0.91)**	<0.001	**0.97 (0.95, 0.99)**	<0.001
5. Gaussian filter	0.043 (0.005)	**0.79 (0.58, 0.90)**	<0.001	**0.83 (0.68, 0.92)**	<0.001
Deep vascular plexus					
Perfusion density					
1. Raw *en face* scan	0.451 (0.018)	**0.90 (0.78, 0.95)**	<0.001	Reference	
2. MBRT filter	0.448 (0.015)	**0.91 (0.81, 0.96)**	<0.001	**0.97 (0.94, 0.99)**	<0.001
3. Hessian filter	0.254 (0.033)	**0.79 (0.56, 0.90)**	<0.001	0.23 (−0.13, 0.54)	0.203
4. Gabor filter	0.456 (0.017)	**0.92 (0.80, 0.96)**	<0.001	**0.97 (0.94, 0.99)**	<0.001
5. Gaussian filter	0.471 (0.017)	**0.90 (0.79, 0.96)**	<0.001	**0.92 (0.83, 0.96)**	<0.001
Vessel density (mm^−1^)					
1. Raw *en face* scan	0.066 (0.003)	**0.82 (0.63, 0.91)**	<0.001	Reference	
2. MBRT filter	0.039 (0.002)	**0.84 (0.68, 0.92)**	<0.001	**0.85 (0.72, 0.93)**	<0.001
3. Hessian filter	0.053 (0.006)	**0.82 (0.63, 0.91)**	<0.001	0.30 (−0.05, 0.59)	0.095
4. Gabor filter	0.058 (0.003)	**0.87 (0.74, 0.94)**	<0.001	**0.95 (0.89, 0.97)**	<0.001
5. Gaussian filter	0.043 (0.003)	**0.85 (0.69, 0.93)**	<0.001	0.50 (0.18, 0.72)	0.004

CI = confidence interval, ICC = intraclass correlation coefficient, MBRT = modified Bayesian residual transform, SD = standard deviation.

Bold interface indicates the filter that provided good‐to‐excellent repeatable scans (ICC > 0.75) and strong correlation to the raw *en face* scan (Pearson's *r* ≥ 0.70).

All the original and filtered 3 × 3‐mm^2^ images yielded excellent to good repeatable scans for the PD and VD metrics in both the vascular layers (ICC > 0.79). Specifically, the use of the MBRT filter yielded the most repeatable SVP metrics (PD: ICC = 0.90; VD: ICC = 0.89). For the DVP, the Gabor filter produced the most repeatable set of PD (ICC = 0.92) and VD (ICC = 0.87) data.

We then examined how well the postprocessed 3 × 3‐mm^2^ images correlated with the original raw *en face* data set. Most of the filters provided PD and VD values that were strongly correlated with the original image (*r* > 0.83). Specifically, images obtained using the Gabor filter had the highest correlation with the original data set for the PD (*r* = 0.97) and VD (*r* = 0.97) of the SVP and the PD (*r* = 0.97) and VD (*r* = 0.95) of the DVP. However, images using the Hessian filter were the least correlated with the original image for all the 3 × 3‐mm^2^ vascular metrics (*r* = 0.23–0.60).

### Mean, repeatability and correlation analysis of 12 × 12‐mm^2^ vascular parameters

The mean PD and VD values of the SVP and DVP of the studied 12 × 12‐mm^2^ scans and their corresponding ICC values are shown in Table 3.

**Table 3:** Mean, intraclass correlation coefficient values and correlation analysis of 12 × 12‐mm^2^ vascular parameters.

Characteristics	A) Mean (SD)	B) ICC (95% CI)	*P* value	C) Pearson's *r* (95% CI)	*P* value
Superficial vascular plexus					
Perfusion density					
1. Raw *en face* scan	0.431 (0.013)	**0.81 (0.61, 0.91)**	<0.001	Reference	
2. MBRT filter	0.449 (0.012)	**0.90 (0.79, 0.95)**	<0.001	**0.89 (0.78, 0.94)**	<0.001
3. Hessian filter	0.257 (0.019)	**0.92 (0.84, 0.96)**	<0.001	**0.78 (0.60, 0.89)**	<0.001
4. Gabor filter	0.424 (0.012)	0.73 (0.45, 0.87)	<0.001	**0.79 (0.60, 0.89)**	<0.001
5. Gaussian filter	0.434 (0.016)	0.54 (0.08, 0.77)	0.015	0.42 (0.09, 0.67)	0.016
Vessel density (mm^−1^)					
1. Raw *en face* scan	0.114 (0.006)	**0.84 (0.67, 0.92)**	<0.001	Reference	
2. MBRT filter	0.056 (0.002)	**0.89 (0.77, 0.94)**	<0.001	**0.89 (0.78, 0.94)**	<0.001
3. Hessian filter	0.073 (0.007)	**0.90 (0.80, 0.95)**	<0.001	**0.92 (0.85, 0.96)**	<0.001
4. Gabor filter	0.083 (0.005)	**0.80 (0.58, 0.90)**	<0.001	**0.97 (0.95, 0.99)**	<0.001
5. Gaussian filter	0.038 (0.002)	0.71 (0.40, 0.86)	<0.001	0.67 (0.42, 0.83)	<0.001
Deep vascular plexus					
Perfusion density					
1. Raw *en face* scan	0.466 (0.016)	0.61 (0.20, 0.81)	0.006	Reference	
2. MBRT filter	0.461 (0.013)	0.73 (0.44, 0.87)	<0.001	**0.87 (0.75, 0.94)**	<0.001
3. Hessian filter	0.164 (0.018)	0.66 (0.31, 0.83)	0.002	0.21 (−0.15, 0.52)	0.250
4. Gabor filter	0.478 (0.015)	0.60 (0.17, 0.80)	0.008	**0.79 (0.61, 0.89)**	<0.001
5. Gaussian filter	0.503 (0.022)	0.56 (0.09, 0.79)	0.013	0.62 (0.35, 0.8)	<0.001
Vessel density (mm^−1^)					
1. Raw *en face* scan	0.143 (0.005)	0.72 (0.42, 0.86)	<0.001	Reference	
2. MBRT filter	0.150 (0.005)	0.75 (0.49, 0.88)	<0.001	**0.92 (0.84, 0.96)**	<0.001
3. Hessian filter	0.055 (0.006)	0.70 (0.40, 0.85)	<0.001	0.45 (0.12, 0.69)	0.010
4. Gabor filter	0.109 (0.005)	0.75 (0.50, 0.88)	<0.001	**0.88 (0.77, 0.94)**	<0.001
5. Gaussian filter	0.047 (0.004)	0.73 (0.45, 0.87)	<0.001	**0.85 (0.71, 0.92)**	<0.001

CI = confidence interval, ICC = intraclass correlation coefficient, MBRT = modified Bayesian residual transform, SD = standard deviation.

Bold interface indicates the filter that provided good‐to‐excellent repeatable scans (ICC > 0.75) and strong correlation to the raw *en face* scan (Pearson's *r* ≥ 0.70).

For repeatability analysis, the original image, MBRT and Hessian filters yielded good‐to‐excellent repeatable SVP metrics (ICC > 0.81). However, the DVP metrics for all images were only moderately repeatable (ICC ≤ 0.75). The data set analysed using the MBRT filter had moderately good ICC values for the analysed vascular metrics: the PD (ICC = 0.90) and VD (ICC = 0.89) of the SVP and the PD (ICC = 0.73) and VD (ICC = 0.75) of the DVP.

Both the MBRT and Gabor filters correlated highly with the original 12 × 12‐mm^2^ scan data set. Perfusion density measurements from the MBRT filter had the highest correlation with the original data set for both the SVP (*r* = 0.89) and DVP (*r* = 0.87). Meanwhile, VD measurements from the Gabor filter had the highest correlation with the original data set for both the plexus (*r* = 0.88–0.97). However, images using the Hessian and Gaussian filters were poorly correlated with the original image for all the 12 × 12‐mm^2^ vascular metrics (*r* = 0.21–0.67).

### Comparison of vascular parameters for a 3 × 3‐mm^2^ to a 12 × 12‐mm^2^ of the same area

**Figure 4:**
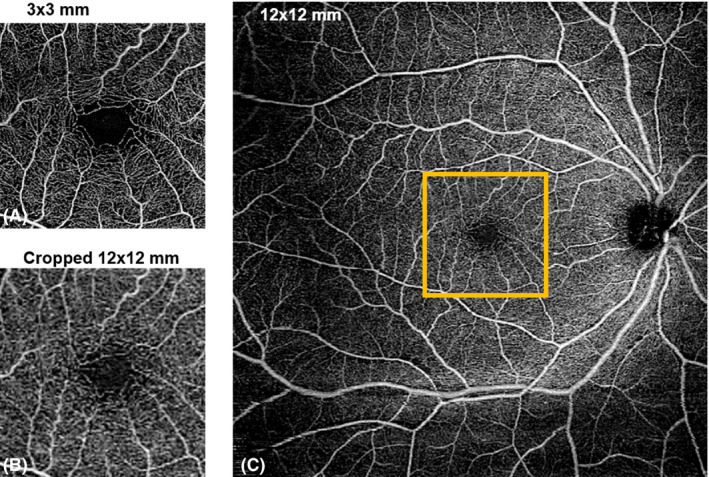
Comparison of A) 3 × 3‐mm^2^ scan, B) cropped 12 × 12‐mm^2^ scan and C) original 12 × 12‐mm^2^ scan of the superficial vascular plexus. A 3 × 3‐mm^2^ area centred on the foveal avascular zone was selected on 12 × 12‐mm^2^ scan (orange box).

The ICCs and Pearson's *r* for the agreement between PD and VD using the two different scan size, 3 × 3‐mm^2^ to a 12 × 12‐mm^2^, are shown in Figure 4 and Table 4. The original image and most of the filters yielded highly repeatable PD values at the SVP layer (ICC > 0.78), except for Hessian filter (ICC = 0.14). However, the ICC of the VD at SVP and DVP measurements was poor (ICC < 0.50). We then examined how well the cropped 12 × 12‐mm^2^ images correlated with the original raw *en face* 3 × 3‐mm^2^ data set. Only images obtained using the MBRT and Gabor filters were moderately correlated with the original data set for the PD (*r* > 0.70) and VD (*r* = 0.73) for the SVP. At the deep capillary plexus, the correlation for PD and VD was poor (*r* < 0.60).

**Table 4:** Comparison of vascular parameters between 3 × 3‐mm^2^ vs cropped 12 × 12‐mm^2^ over the same region.

Characteristics	A) Mean (SD) 3 × 3‐mm^2^	B) Mean (SD) Cropped 12 × 12‐mm^2^	C) *P* value*	D) ICC (95% CI)	*P* value	E) Pearson's r (95% CI)	*P* value
Superficial vascular plexus							
Perfusion density							
1. Raw *en face* scan	0.449 (0.018)	0.451 (0.014)	0.664	**0.82 (0.63, 0.91)**	<0.001	Reference	
2. MBRT filter	0.452 (0.015)	0.454 (0.013)	0.633	**0.78 (0.54, 0.89)**	<0.001	**0.75 (0.54, 0.87)**	<0.001
3. Hessian filter	0.298 (0.037)	0.158 (0.027)	<0.001	0.14 (0, 0.47)	<0.001	0.61 (0.34, 0.79)	<0.001
4. Gabor filter	0.451 (0.021)	0.450 (0.015)	0.393	**0.84 (0.67, 0.92)**	<0.001	**0.70 (0.46, 0.84)**	<0.001
5. Gaussian filter	0.444 (0.024)	0.448 (0.016)	0.282	**0.86 (0.72, 0.93)**	<0.001	0.65 (0.39, 0.82)	<0.001
Vessel density (mm^−1^)							
1. Raw *en face* scan	0.066 (0.004)	0.035 (0.002)	<0.001	0.02 (0, 0.12)	<0.001	Reference	
2. MBRT filter	0.038 (0.001)	0.050 (0.002)	<0.001	0.01 (0, 0.06)	0.002	**0.77 (0.58, 0.88)**	<0.001
3. Hessian filter	0.060 (0.006)	0.021 (0.004)	<0.001	0.05 (0, 0.21)	<0.001	0.62 (0.34, 0.80)	<0.001
4. Gabor filter	0.059 (0.004)	0.034 (0.002)	<0.001	0.05 (0, 0.21)	<0.001	**0.73 (0.51, 0.86)**	<0.001
5. Gaussian filter	0.043 (0.005)	0.030 (0.002)	<0.001	0.17 (0, 0.53)	<0.001	**0.72 (0.50, 0.85)**	<0.001
Deep vascular plexus							
Perfusion density							
1. Raw *en face* scan	0.451 (0.018)	0.469 (0.011)	<0.001	0.15 (0, 0.49)	<0.001	Reference	
2. MBRT filter	0.448 (0.015)	0.465 (0.010)	<0.001	0.15 (0, 0.49)	<0.001	0.61 (0.33, 0.79)	<0.001
3. Hessian filter	0.254 (0.033)	0.076 (0.019)	<0.001	0.07 (0, 0.28)	<0.001	−0.17 (−0.49, 0.19)	0.353
4. Gabor filter	0.456 (0.017)	0.469 (0.011)	<0.001	0.18 (0, 0.53)	<0.001	0.63 (0.36, 0.80)	<0.001
5. Gaussian filter	0.471 (0.017)	0.476 (0.013)	<0.001	0.39 (0, 0.75)	<0.001	0.60 (0.32, 0.79)	<0.001
Vessel density (mm^−1^)							
1. Raw *en face* scan	0.066 (0.003)	0.037 (0.001)	<0.001	0.01 (0, 0.03)	0.071	Reference	
2. MBRT filter	0.039 (0.002)	0.053 (0.001)	<0.001	0 (0, 0.03)	0.064	0.39 (0.04, 0.65)	0.029
3. Hessian filter	0.053 (0.006)	0.010 (0.002)	<0.001	0.02 (0, 0.1)	<0.001	−0.28 (−0.57, 0.08)	0.124
4. Gabor filter	0.058 (0.003)	0.036 (0.001)	<0.001	0.01 (0, 0.07)	0.034	0.34 (−0.01, 0.61)	0.058
5. Gaussian filter	0.043 (0.003)	0.033 (0.001)	<0.001	0.1 (0, 0.38)	<0.001	0.30 (−0.05, 0.59)	0.090

CI = confidence interval, ICC = intraclass correlation coefficient, MBRT = modified Bayesian residual transform, SD = standard deviation.

Bold interface indicates the filter that provided good‐to‐excellent repeatable scans (ICC > 0.75) and strong correlation to the raw *en face* scan (Pearson's *r* ≥ 0.70).

* Paired sample *t* test used to analyse the differences between 3 × 3‐mm^2^ and cropped 12 × 12‐mm^2^ over the similar region.

## Discussion

Current literatures on quantitative OCTA metrics have employed software algorithms proprietary to their respective OCTA devices (Fenner et al. 2018) or use Frangi (Camino et al. 2016; Ting et al. 2017) or Gabor filters (Hendargo et al. 2013) for postimage processing. However, none have compared metrics obtained using different postimage processing algorithms. Our findings show that scans taken by the SS‐OCTA prototype exhibit good‐to‐excellent repeatability, with variations in the repeatability of OCTA vascular metrics derived from different scan dimensions and postimage processing methods. Postprocessing has an effect on the repeatability and the OCTA numerical values, suggesting the need for caution when comparing results across studies that use different instrumentations.

Differences in PD and VD values mainly arose from the detection and quantification of smaller vessels such as capillaries (Figures 1 and 2). While larger vessels have a higher signal‐to‐noise ratio and were thus consistently imaged, capillaries have a lower signal‐to‐noise ratio and smaller calibre. This issue is further complicated in 12 × 12‐mm^2^ scans, where the peripheral retina is less well‐illuminated compared with the macula. To overcome this challenge, different filters are often applied to OCTA scans to enhance these finer features (Chu et al. 2016; Iafe et al. 2016). We also recognize that no amount of filtering process can fully mitigate these issues. This is clearly illustrated in the comparison between the 3 × 3‐mm^2^ and a 12 × 12‐mm^2^ of the same area (Figure 4). There remain two possibilities why small capillaries are more difficult to image when using a 12 × 12‐mm^2^ scanning protocol than a 3 × 3‐mm^2^. First, the issue of lateral oversampling: each A‐Scan essentially illuminates a spot of around 20 *μ*m diameter. Hence, any structures that reflect light, even in the case of a 6 *μ*m capillary, show up as bright pixels. On a 3 × 3‐mm^2^ scan, with a sampling spacing of only 10 *μ*m, those A‐Scan spot sizes truly overlap; hence, every feature gets illuminated and reflects light back, translating to better detection and representation of retinal capillaries. However, with a 12 × 12‐mm^2^ scan, the sampling spacing is 24 *μ*m; hence, small areas inbetween two A‐Scans are not illuminated, which means small capillaries could go unnoticed. The second issue relates to the number of B‐scans. In the 3 × 3‐mm^2^ scan, it calculates the variation out of 4 B‐scans per line, which again should capture weaker signals, thereby increasing its sensitivity and reducing the noise in the signal, which translates to a higher quality image. However, with a 12 × 12‐mm^2^ scan, it calculates the variation out of 2 B‐scans per line only.

On comparison of the different filters, the MBRT filter consistently yielded highly repeatable PD and VD measurements from the SVP and DVP of both 3 × 3‐mm^2^ and 12 × 12‐mm^2^ scans. This finding is mainly because the MBRT filter can enhance the smaller vascular structures (Figures 1B, 2B) compared to the corresponding raw *en face* images (Figures 1A, 2A) and is sensitive to vascular structures even in the poorly illuminated peripheral retina of 12 × 12‐mm^2^ scans (Figure 2B). However, a downside of the MBRT filter may be the slightly longer processing time of approximately 2.5 seconds, double that of the Gabor filter.

Interestingly, other filters were more suited for different scan dimensions. The Gaussian filter obtained repeatable vascular metrics from 3 × 3‐mm^2^ scans but was less repeatable for 12 × 12‐mm^2^ scans. While these filters enhanced small capillaries well (Figures 1D, 1E, 2D, 2E), they were unable to account for the lower illuminance of the peripheral retina in 12 × 12‐mm^2^ scans (Figures 2D, 2E). In contrast, while the Hessian filter fared poorly for 3 × 3‐mm^2^ scans due to its inability to enhance small vessel structures as well (Figure 1C), it performed the best for 12 × 12‐mm^2^ scans due to its lower sensitivity to variations in illuminance in these widefield OCTA scans (Figure 2C).

We also showed that the 4 filters, when applied to the same OCTA scan, correlated differently with the original *en face* image. An example of such a great variation is the vessel density of the DVP, as obtained from the 3 × 3‐mm^2^ scan (*r* ranged from 0.30 to 0.95). This disparity signified that the direct comparison of the OCTA metrics between different studies may be challenging if different postprocessing methods were used. Additionally, a poor correlation with the original *en face* image may suggest data loss or distortion, in which case will compromise the discriminative power of the poorly correlated filters. Further studies are required to comprehensively compare the discriminative ability of different postimage processing methods.

Given its high repeatability, association with disease severity, ease‐of‐use and non‐invasive nature, OCTA has potential in longitudinally assessing various patient populations to identify disease progression. However, this recommendation is made with a few caveats. Firstly, a repeatable postimage processing method must be identified and comprehensively described for future clinical studies to replicate if interested. Secondly, all scans must be aligned to ensure that the same vascular regions are assessed in each repeated scan as it is difficult to consistently imaging the same area. Figure 3 shows that two scans of the same eye (3A, 3B) may sample different vessels due to variances in alignment (3C). However, this issue can be addressed by co‐localizing similar areas for disease monitoring (3D). However, a consequence of postimaging alignment is that a smaller area than the original scan protocol is monitored over time. A possible solution to overcome this issue is to use a larger scan dimension. For example, the macula could be imaged using a 6 × 6‐mm^2^ scan instead such that the overlapping regions are of significant size. However, increasing the scan area may compromise sampling resolution or increase acquisition time, making scans more prone to motion artefacts.

For 3 × 3‐mm^2^ OCTA scans, the association between poor control of systemic diseases, such as diabetes mellitus (Ting et al. 2017) and hypertension (Chua et al. 2018a,b,^2019^), and reduced capillary density in both the SVP and DVP is well studied. Additionally, given the high repeatability of 3 × 3‐mm^2^ OCTA vascular metrics in both the SVP and DVP, there is utility in using 3 × 3‐mm^2^ OCTA scans to longitudinally assess for disease progression when indicated. This recommendation is supported by earlier studies that report high ICC values of VD measurements in the SVP of 3 × 3‐mm^2^ SD‐OCTA scans in different patient populations, including healthy participants (ICC = 0.73) (Coscas et al. 2016), diabetic patients (ICC = 0.97) (Czako et al. 2018) and those with varied retinal diseases (ICC = 0.81) (Lee et al. 2019).

**Figure 5:**
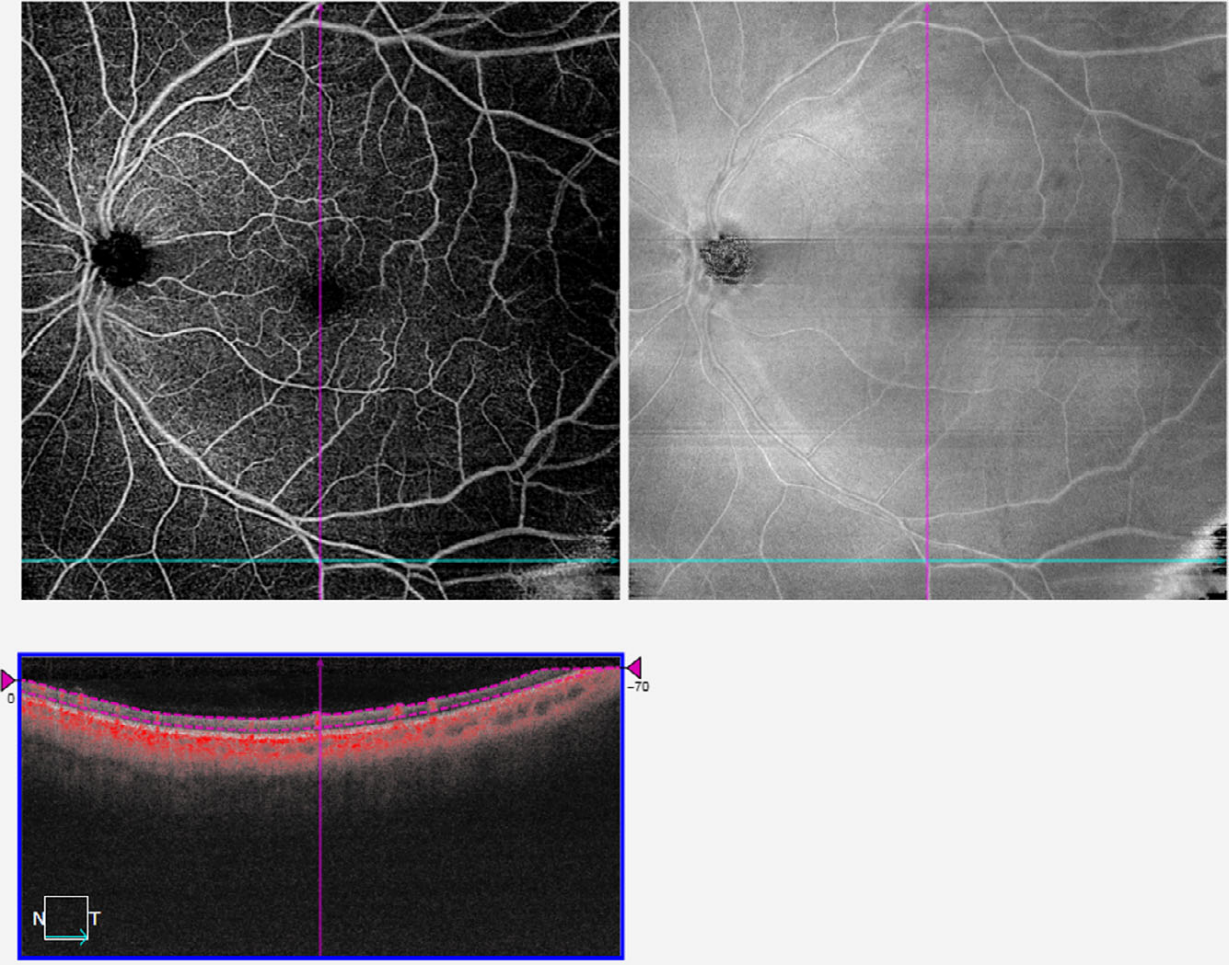
Data loss at the inferotemporal region due to the area being out of the scan (see B‐scan).

**Figure 6:**
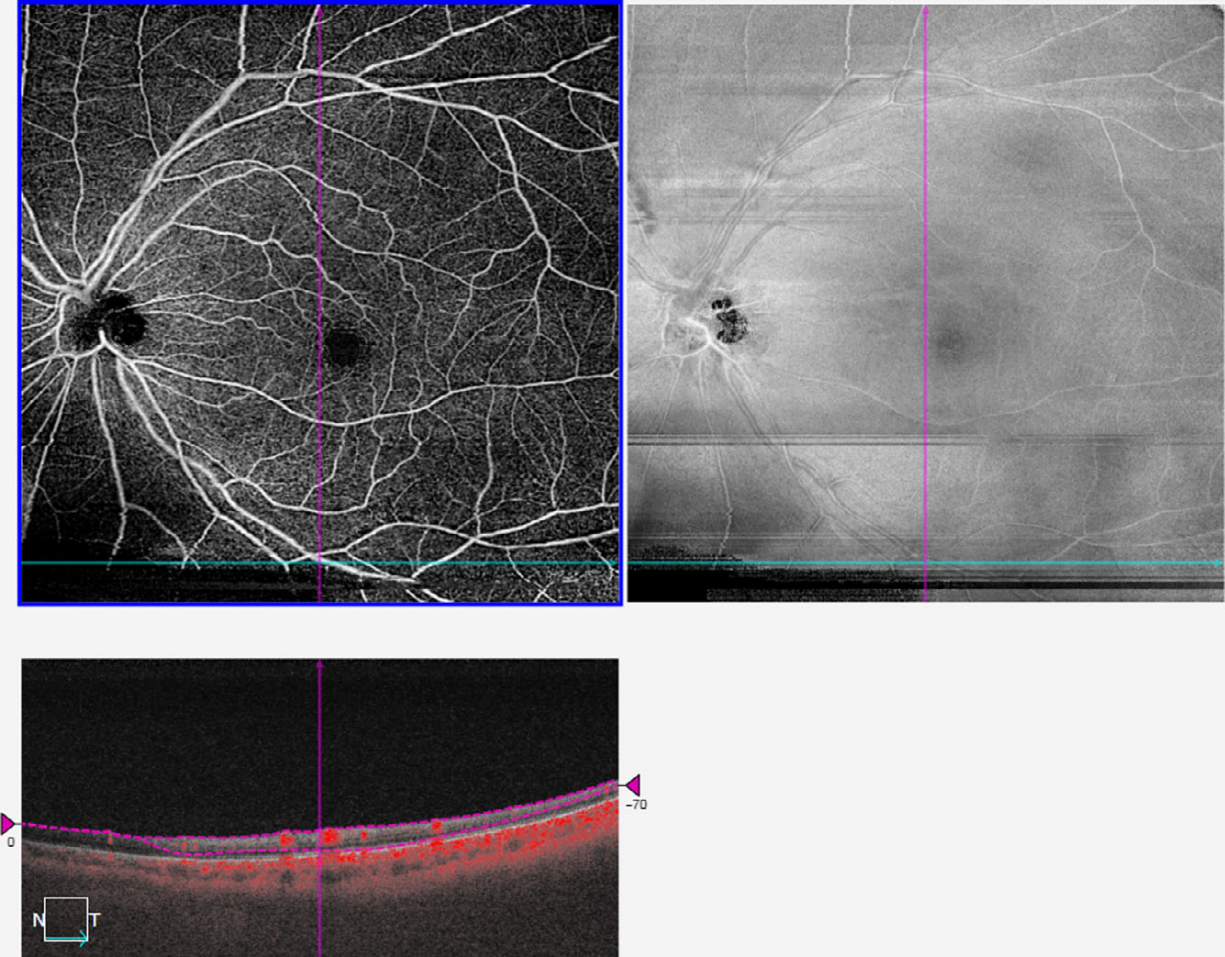
Data loss at the inferior retina due to segmentation errors arising from signal loss (see B‐scan).

While 3 × 3‐mm^2^ scans are valuable in providing high‐resolution structural and angiographic images of the macula, it is also crucial to visualize peripheral regions of the retina as they may be implicated in other ocular diseases. In patients with diabetic retinopathy, 12 × 12‐mm^2^ scans can evaluate the peripheral retina for capillary dropout and neovascularization. Furthermore, the depth‐resolved nature of OCTA allows it to study the DVP, an early site of damage in diabetic retinopathy (Moore et al. 1999). In patients with glaucoma, studies have shown that reduced macular (Yarmohammadi et al. 2017), circumpapillary (Lommatzsch et al. 2018) and whole‐image VD of 4.5 × 4.5‐mm^2^ scans on OCTA (Yarmohammadi et al. 2016) are significantly associated with disease severity. As a relatively new scan protocol, widefield 12 × 12‐mm^2^ scans are especially valuable in glaucoma diagnostics and management as encompass the macular and circumpapillary vasculature in one single image (Wu et al. 2018). Despite the lack of existing literature discussing the repeatability of 12 × 12‐mm^2^ scans, our results support the longitudinal use of 12 × 12‐mm^2^ scans in managing patients with diabetic retinopathy and glaucoma. However, there are problems intrinsic to the sheer area of imaging (Ang et al. 2019), such as the peripheral retina being out of bounds in eyes with poorly centred scans (Figure 5) or scans with low signal in the peripheries also known as vignetting, leading to segmentation errors (Figure 6). Widefield OCT imaging is much more sensitive to these artefacts, which are the result of misalignments, where the lateral position of the scan pivot of an OCT retinal scanner has not been imaged to the centre of the ocular pupil (Carrasco‐Zevallos et al. 2015). These acquisition effects can hinder the reproducibility of OCTA metrics, and no filters are able to compensate for those shortcomings. Possible solutions would be to crop out the peripheral retina if such issues arise or based on the corresponding structural images, although a balance must be struck between diagnostic accuracy and maximizing scan inclusion.

### Study Strengths and Limitations

The current study is based on data obtained in an older cohort, which makes it clinically more applicable as eye diseases often disproportionately affect the elderly. However, the generalizability of our findings may be limited as this study was conducted in a single centre with a small sample size. Future studies should be conducted with a larger sample size in multiple centres to more reliably evaluate the repeatability and generalizability of OCTA findings.

In conclusion, our findings show that PD and VD metrics in the SVP and DVP of both 3 × 3‐mm^2^ and 12 × 12‐mm^2^ scans exhibit good repeatability, with variations in the repeatability of OCTA vascular metrics derived from different postimage processing methods. We therefore advocate uniformity in the postprocessing methods for the purpose of enhancing accuracy when comparing retinal vascular abnormalities in patients with retinal diseases.
